# The Optimal Approach to Surgical Management of Goblet Cell Carcinoid of the Appendix: A Systematic Review

**DOI:** 10.3390/diagnostics14161773

**Published:** 2024-08-14

**Authors:** Mahmoud M. Salama, Maeve A. O’Neill, Éanna J. Ryan, Niall J. O’Sullivan, Timothy S. Nugent, Hugo C. Temperley, Brian J. Mehigan, John O. Larkin, David Gallagher, Grainne O’Kane, Paul H. McCormick, Michael E. Kelly

**Affiliations:** 1 Department of Surgery, St. James’s Hospital, D08 NHY1 Dublin, Ireland; salamam@tcd.ie (M.M.S.);; 2 School of Medicine, University of Galway, H91T K33 Galway, Ireland; 3 School of Medicine, Trinity College Dublin, D02 PN40 Dublin, Ireland; 4 Trinity St. James’s Cancer Institute, D08 NHY1 Dublin, Ireland

**Keywords:** goblet cell carcinoid, appendiceal neoplasm, appendicectomy, hemicolectomy, survival analysis

## Abstract

Introduction: Goblet cell carcinoid (GCC) is a rare and poorly understood appendiceal neoplasm, exhibiting mixed histological and aggressive clinical features. Current guidelines recommend right hemicolectomy in all cases, although there is conflicting evidence that appendicectomy alone may be sufficient. This review aims to identify the optimal surgical management for appendiceal GCC. Methods: A systematic review was performed by searching MEDLINE, Embase, Scopus and the Cochrane Register of Controlled Trials. Randomised controlled trials, cohort studies or large case series (>5 patients) reporting clinical outcomes for patients undergoing surgical management of GCC of the appendix were included. Outcomes extracted included participant and tumour characteristics, type of surgery and survival data. Results: A total of 1341 studies were retrieved. After duplicate removal, 796 titles were screened for relevance prior to abstract and full text review. A total of six studies were included for analysis, comprising 3177 patients—1629 females and 1548 males. The median age ranged from 51 to 72 years. A total of 2329 patients underwent right hemicolectomy, while 824 were treated with appendicectomy only. Overall, the included studies report increased survival in patients undergoing right hemicolectomy compared to appendicectomy alone. A meta-analysis was not possible due to insufficient data reported in the published literature to date. Conclusions: There is no consensus regarding the optimal surgical management of appendiceal GCC, as outcomes-based data comparing surgical interventions are lacking. It is possible that some patients with favourable features are overtreated. The absence of robust evidence to support a more conservative approach means that right hemicolectomy remains the standard of care for all patients, in keeping with current international guidelines. The rarity of this condition and limited data in the published studies remain barriers to evidence-based best clinical practice.

## 1. Introduction

Goblet cell carcinoid (GCC) is a rare tumour type occurring almost exclusively in the vermiform appendix [1]. These neoplasms express both mucinous and neuroendocrine differentiation, and are composed primarily of goblet cells with occasional neuroendocrine and Paneth cells [2]. Histologically, cells are arranged as discrete nests arising in the lamina propria, with concentric involvement of the appendiceal wall [3]. GCC is a rare subtype of mixed adenoneuroendocrine carcinomas (MANEC) and is considered a separate, more aggressive entity than a standard appendiceal carcinoid tumour [4]. Identified in approximately 0.3–0.9% of appendicectomies, GCC represents less than 14% of malignant tumours of the appendix [4,5]. While GCC is almost unique to the appendix, it has also been described in other regions of the gastrointestinal tract [6]. Diagnosis is often incidental [5].

The classification and grading of GCC present a particular challenge, as these tumours exhibit classic features of both adenocarcinomas and neuroendocrine tumours [2]. The distinctive histology and variable behaviour of GCC have generated debate amongst pathologists and surgeons [1]. Accurate characterisation is essential in order to predict prognosis and guide further management [7]. Historically considered a subtype of carcinoid tumour, current evidence suggests that GCC is a distinct histological entity arising from multipotent epithelial crypt stem cells as opposed to the subepithelial neuroendocrine cells from which classic carcinoid tumours are derived [8]. While the histopathological classification and biological behaviour of appendiceal GCC remain uncertain, it is generally accepted that they are more aggressive than simple appendiceal carcinoids [9], with a reported 5-year overall survival of 40–75% [5,10].

Given the rarity and limited understanding of appendiceal GCC, there is no clear international consensus regarding its optimal management [5]. The North American Neuroendocrine Tumour Society (NANETS) and European Neuroendocrine Tumour Society (ENETS) both advocate for right hemicolectomy in all patients found to have GCC [11,12]. However, previous studies have suggested that appendicectomy alone may be sufficient [10]. Given the rarity of this tumour subtype, there is a paucity of studies in the literature reporting clinical outcomes of GCC. The existing evidence is derived from small, single-centre retrospective studies. A randomised controlled trial comparing treatment methods is unlikely to ever be published [10].

The aim of this review was to assess the current evidence base and identify the optimal surgical management of appendiceal GCC. Specifically, we aimed to compare right hemicolectomy versus appendicectomy, evaluating the primary outcome measures of disease-free survival and overall survival resulting from each intervention.

## 2. Methods

This systematic review was conducted in accordance with the Preferred Reporting Items for Systematic Reviews and Meta-Analyses [13] guidelines. Local institutional ethical approval was not required.

### 2.1. Search Strategy

An electronic search of Embase, PubMed/MEDLINE, Scopus and the Cochrane Register of Controlled Trials databases was performed. The final search was performed on 26th May 2024. References of all included publications were also screened. The search was performed by two independent reviewers (MMS and MON) using a predetermined search strategy. Included studies were limited to papers published after the year 2000 and in English.

### 2.2. Inclusion and Exclusion Criteria

Randomised controlled trials, cohort studies and large case series (>5 patients) comparing appendectomy vs. right hemicolectomy for goblet cell carcinoma of the appendix were included. All studies included patients aged >18 years at the time of diagnosis. Studies including patients with metastatic or peritoneal disease were excluded, unless outcomes were reported separately.

### 2.3. Study Selection

Duplicate studies were manually removed before two authors (MMS and MON) independently screened all titles for relevance using COVIDENCE software [14]. For studies considered potentially relevant, abstracts and full texts were reviewed for eligibility using the inclusion and exclusion criteria outlined above. Disagreements were resolved by discussion until a final verdict was reached, with final arbitration from the senior author (MK).

### 2.4. Data Extraction and Quality Assessment

Following final paper selection, data were extracted from the included studies and collated using Microsoft Excel (version16.86) [15]. Risk of bias was assessed using the Newcastle–Ottawa Scale [16].

### 2.5. Outcomes

Collected outcomes included first author name, year of publication, study design, country of origin, number of patients with GCC, number of patients undergoing each surgical intervention (appendicectomy/right hemicolectomy), median age (and range) at diagnosis, male-to-female ratio, clinicopathological data (tumour location, tumour stage, tumour grade, Tang classification and tumour size) and survival outcomes (overall survival at the end of the study period and recurrence rates; follow-up period was included if reported by the paper).

## 3. Results

### 3.1. Literature Search

**Figure 1:**
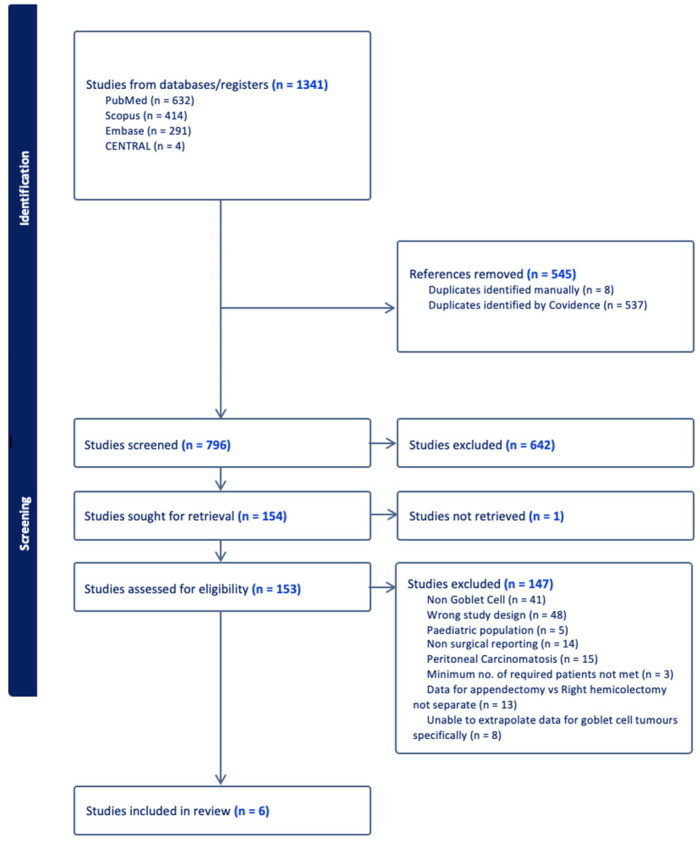
PRISMA flowchart of study selection process.

A total of 1341 studies were retrieved from the electronic literature search as detailed above. After the removal of 545 duplicates, the remaining 796 titles were screened for relevance before a review of the abstracts and full text manuscripts of potentially relevant studies for eligibility. After final full paper review, six studies fulfilled the predetermined inclusion criteria and were included for analysis (Figure 1).

### 3.2. Study Characteristics

The six included studies comprised four retrospective cohort studies [17,18,19,20,21] and two retrospective registry reviews [22,23]. The studies originated from the USA [19,22,23], UK [20], Canada [17,21] and Switzerland [17,21]. Four of the included studies were published after 2010 (Table 1).

### 3.3. Patient Demographics

A total of 3177 patients with primary appendiceal GCC were included. These included 1629 females and 1548 males. Median age reported in these studies ranged from 51 to 72 years. One study reported on mean age [22] and another only divided patients into two broad categories—above or below 65 years of age [23].

### 3.4. Tumour Characteristics

Only two studies reported on tumour location [17,20]. These studies had missing data for 10% of patients with respect to tumour location. Of the 25 patients with tumour locations reported, the most common location was in the appendiceal base (48%), followed by the appendix tip (28%) and finally the appendix body (24%).

Tumour size was available in two studies, including 93 patients in total [17,18,21]. The smallest size reported was 0.7 cm [17], while the largest was 3 cm [17]. One study included data on the number of patients with tumours of varying sizes [18]. Of these, 90.5% of patients had tumours less than 2 cm in size. Tumour stage was reported for 3068 patients in three studies [18,20,22,23]. Stage II disease was the most common overall, accounting for 64.8% of the entire cohort. Stage IV represented the least common stage in the overall cohort, accounting for 6.4% of patients.

Tumour grade was reported in three studies, with data available for a total of 1490 patients [20,22,23]. Grade 1 tumours were the most common overall, accounting for 43.6% of cases. This was followed by Grade 2 in 33.1% and Grade 3 in 23.3% of cases.

Tang Classification was highlighted in two studies, including 100 patients [12,13]. This is a histopathological classification categorizing GCC into ‘typical’, ‘signet ring’ or ‘poorly differentiated’ morphological subtypes—Tang A, B and C respectively. Tang A accounted for the majority of cases in the included studies, with 52.7%. Tang B and Tang C accounted for 36.7% and 10.7%, respectively.

Lymph node involvement was examined in several studies [17,19,21,22,23]. However, as not all patients underwent formal right hemicolectomy and some studies had missing data, nodal status was available for 2942 patients. Of these, 588 patients had histologically confirmed node-positive disease (20%).

### 3.5. Operative Details

Combining all the included studies, 2329 patients underwent right hemicolectomy, while 824 patients were treated with appendicectomy only (Table 2). Data specifically detailing index procedure and completion operation—whether right hemicolectomy was performed upfront or following an initial appendicectomy—were available in four studies, including 124 patients [17,18,19,20,21].

The two papers that did not differentiate between index and completion surgery were the two large population-based registry review studies [22,23]. These included a total of 3053 patients, of whom 798 underwent appendicectomy alone and 2255 underwent right hemicolectomy.

From studies providing data on the index procedure performed, the most common index surgical procedure across all studies was appendicectomy in 96 patients. Other index procedures included right hemicolectomy in 8 cases, ileocolic resection in 1, cytoreductive surgery in 10 and unspecified in 8. Following initial appendicectomy, 70 patients went on to have a subsequent completion procedure, with right hemicolectomy the procedure of choice in 66 cases. Two patients instead underwent ileocolic resection, and the remaining two patients required multi-visceral cytoreductive surgery.

### 3.6. Survival

Six studies reported survival data for appendicectomy versus right hemicolectomy [17,18,19,20,21,22]. Overall, the included studies reported increased survival in patients undergoing right hemicolectomy compared to appendicectomy alone.

Against the overall trend, two small retrospective cohort studies reported that appendicectomy was associated with higher survival than hemicolectomy [5,11]. It should be noted that the numbers in each group were small. With a combined total of just four patients undergoing appendicectomy alone, both studies reported 100% survival in the appendicectomy group (Table 3).

For one large registry review comprising 1970 patients, it was not possible to extrapolate individual patient data with respect to tumour characteristics, surgical management and outcomes [23]. Pooled patient data from this study demonstrated improved survival for patients undergoing right hemicolectomy, and on subgroup analysis based on tumour stage, survival reached statistical significance only for patients with stage II disease.

A second large registry review comprising 1083 patients also reported improved survival for patients with GCC undergoing right hemicolectomy compared to appendicectomy. This survival advantage was seen only for patients with T3/T4 tumours (85.4% vs. 82%, *p* = 0.028). In contrast, for patients with T1/T2 disease, similar 5-year survival was observed whether patients underwent appendicectomy alone or formal right hemicolectomy (83.6% vs. 87.3%, *p* = 0.176).

### 3.7. Assessment of Bias

Risk of bias was assessed using the Newcastle–Ottawa Scale. All of the included studies were deemed to be of fair quality (Table 4).

## 4. Discussion

This systematic review of the available literature provides an up-to-date overview of the surgical management of appendiceal GCC. We highlight the observed patterns relating to various tumour characteristics, the operative treatment strategies employed and the associated survival outcomes for patients undergoing surgical management of this rare entity. It has previously been postulated that appendicectomy alone may be sufficient for patients with early-stage, low-grade GCC with clear surgical margins and no nodal involvement [24,25,26], although a solid evidence basis to support this approach remains lacking. We describe current practices regarding the definitive surgical management of patients with non-metastatic appendiceal GCC and compare the reported survival outcomes for patients undergoing right hemicolectomy versus appendicectomy only.

We observed a relative scarcity of studies in the literature relating to the surgical management and survival outcomes of patients with GCC of the appendix. Most of the available literature on the subject of appendiceal GCC relates predominantly to clinicopathological features and overall survival trends rather than comparisons of different surgical interventions and their associated outcomes, as was the focus of our study. Most of the eligible studies were small, single-centre, retrospective reviews, with a combined total of only 124 patients from four studies and the remaining two large population-based registry reviews contributing the majority of the patients. The paucity of available studies and the small cohort sizes in the existing studies both reflect the rarity of this tumour type and contribute to the persistent void in knowledge surrounding their optimal management. In addition to limited studies with small sample sizes, the heterogeneity of these studies, missing data and variance in outcome reporting combine to preclude a high-quality statistical analysis of these data. A lack of similar follow-up periods, median overall survival and uncertainty intervals prevented any meaningful statistical analysis from being carried out. It is therefore difficult to draw meaningful conclusions, although this is nonetheless the best available evidence to guide the management of patients diagnosed with this rare tumour.

Currently, treatment decisions for patients with appendiceal GCC are made with reference to two international guidelines—the 2013 ENETS Consensus Guidelines for the Management of Patients with Neuroendocrine Neoplasms from the Jejuno-Ileum and the Appendix including Goblet Cell Carcinomas, and the 2019 American Society of Colon and Rectal Surgeons Clinical Practice Guidelines for the Management of Appendiceal Neoplasms. According to the American guidelines, while LAMN and appendiceal NET can be safely treated with appendicectomy alone in the setting of favourable histopathological features, patients with non-metastatic adenocarcinoma of the appendix should undergo right hemicolectomy. GCC is included in this category with the same recommendation applied due to its observed similarity to high-grade appendiceal neoplasms. This is a strong recommendation based on low-quality evidence (grade 1C). The European guidelines, based on an expert consensus, also recommend oncological right hemicolectomy as the standard of care for patients with non-metastatic appendiceal GCC due to the risk of nodal metastases and the associated poor prognosis for patients who develop metastatic disease as a result of this tumour.

Our review demonstrates that right hemicolectomy was favoured as the definitive surgical procedure in 73.3% of all cases. However, perhaps surprisingly, given the agreement of the published guidelines both advocating right hemicolectomy, a significant proportion of patients were managed with appendicectomy alone (25.9%). It is worth noting that all of the studies included for analysis in our systematic review report data for the surgical management of patients predating the 2019 guideline. All studies also include a proportion of patients treated prior to the 2013 guideline. Treatment decisions at that time were therefore likely based on established practices for other appendiceal neoplasms, such as adenocarcinoma or typical carcinoids, and were possibly influenced by an understanding that GCCs may represent a more aggressive histological subtype, as described in the pre-existing literature focusing on histopathological classification and clinical behaviour.

Overall, our included studies reported increased survival in patients with non-metastatic GCC undergoing right hemicolectomy compared to appendicectomy alone. The greatest level of evidence comes from the two large registry reviews, which encompass just over 3000 patients between them. Both of these studies report enhanced overall survival for patients with GCC undergoing right hemicolectomy compared to appendicectomy. For one of these studies, subgroup analysis was performed based on tumour stage, with the finding that although a survival benefit was observed for all stages, this reached statistical significance only for patients with stage II disease. The other large registry review demonstrated a survival advantage for patients with T3/T4 tumours undergoing right hemicolectomy versus appendicectomy alone, while no difference in 5-year survival was observed for patients with T1/T2 disease undergoing either surgical intervention as definitive management. These findings are in keeping with previously held assumptions that early-stage disease may be suitable for the omission of right hemicolectomy [27,28]; however, each in isolation falls short of providing evidence to support the safety of implementing this approach in clinical practice. Other studies have failed to show any benefit from right hemicolectomy in terms of overall and recurrence-free survival [29], which highlights that there is likely a sub-cohort of patients with GCC who are likely adequately managed with appendectomy alone.

We acknowledge several limitations of our review. Due to the large volume of missing data and heterogeneity in reporting, we were unable to assess the contribution of tumour stage and grade to the survival of patients undergoing surgical management of GCC. Histological classification is of known clinical importance, as it has been shown to correlate with prognosis, with higher-grade disease associated with reduced disease-specific survival [30]. It has been previously suggested that tumour grade should be taken into consideration when determining the extent of surgical resection, although there remains no convincing evidence to justify this approach [30]. While there are published studies reporting survival outcomes for various stages and histological grades of GCC, there is limited evidence of clinical outcomes relating to these prognostic features in the context of comparing surgical interventions.

Our review included two large registry reviews for analysis. While these studies were both developed using the US population-based National Cancer Database, each used separate search strategies and had different inclusion criteria and varying timeframes for the recruitment of patients. It is, however, likely that there may be some overlap between these patient cohorts. Ultimately, there are a considerable lack of high-quality, evidence-based studies in the literature to guide the management of patients with appendiceal GCC. All of our included studies comprised level 3 evidence, with no higher level of evidence currently published on this topic.

Our search highlighted that several cancer databases with information regarding GCC exist. While we included two studies that analysed the US population-based “National Cancer database”, other studies such as that by Palmer et al. used the “English National Cancer Registration and Analysis Service database” [31]. Appropriate data collection and follow-up of patients in these registries will pave the way for an analysis of whether either of these procedures allow for evidence-based surgical recommendations, although this has yet to be realised due to the inadequate follow-up periods and incomplete data entry in these databases. As more institutions continue to digitize patient records, which makes information more easily accessible, we hope that more studies highlighting outcomes regrading appendectomy versus hemicolectomy in this rare entity will be published to allow for additional analyses and an evidence-based approach to management. Nonetheless, our study provides a summary of current outcomes in the literature comparing both procedures.

## 5. Conclusions

There remains no clear consensus regarding the optimal surgical management of appendiceal GCC, as outcomes-based data comparing surgical interventions are lacking. Current data are insufficient to recommend one procedure over the other. As such, right hemicolectomy remains the standard of care for all patients, in keeping with current international guidelines.

## Figures and Tables

**Table 1:** Study Characteristics.

Study	Country	Study Type	No. of GCC pts
Bucher 2005 [17]	Switzerland	Retrospective Cohort	7
Byrn 2006 [19]	USA	Retrospective Cohort	10
Clift 2018 [20]	UK	Retrospective Cohort	21
Tsang 2018 [21]	Canada	Retrospective Cohort	86
Kowalsky 2021 [22]	USA	Retrospective Registry Review	1083
Marks 2023 [23]	USA	Retrospective Registry Review	1970

**Table 2:** Surgical intervention breakdown.

Study	Appendectomy Only	Hemicolectomy
Bucher 2005 [17]	3	3
Byrn 2006 [19]	1	5
Clift 2018 [20] *	6 *	15 *
Tsang 2018 [21]	16	51
Kowalsky 2021 [22]	197	886
Marks 2023 [23]	601	1369
Total	824	2329

*, data on metastatic disease could not be excluded.

**Table 3:** Survival in patients undergoing appendectomy vs. hemicolectomy.

Study	Survival and Recurrence	Appendectomy	Hemicolectomy
Bucher 2005 [17]	Survival	100% [median follow-up of 84 mths]	66% [median follow-up of 60 mths]
	Recurrence	0	0
Byrn 2006 [19]	Survival	100% [5 mths]	80% [2 mths–2 yrs]
	Recurrence	0	4.2%
Clift 2018 [20] *	Survival	50% [median follow-up of 23 mths]	87.5% [median follow-up of 34 mths]
	Recurrence	-	-
Tsang 2018 [21]	Survival	77% [5 yr OS: Stage I–III]	90% [5 yr OS: Stage I–III]
	Recurrence	62% [5 yr RFS]	79% [5 yr RFS]
Kowalsky 2021 [22]	Survival	82.8% [5 yr OS: Stage I + II]	85.9% [5 yr OS: Stage I + II]
	Recurrence	-	-
Marks 2023 [23] **	Survival		-
	Recurrence	-	-

*, data on metastatic/stage IV could not be excluded; **, no survival data, but risk-adjusted survival analysis demonstrated that RHC showed statistically significant overall survival compared to simple appendectomy in stage II disease only; OS, overall survival; RFS, recurrence-free survival; mths, months.

**Table 4:** Newcastle–Ottawa Scale: Assessment of bias in included studies.

Cohort Studies	Selection				Comparability	Outcome			Total
	Representativeness of the Exposed Cohort	Selection of the Non-exposed Cohort	Ascertainment of Expsoure	Demosntartion that Outcome of Intesrest Was Not Present at the Start of the Study	Comparability of Cohorts on the basisof design or analysis	Assessment of Outcome	Was Follow Up Long Enough for Outcomes to Occur ^	Adaquacy of Follow Up of Cohorts	
Bucher 2005 [17]	*	*	*	0	0	*	0	*	5
Byrn 2006 [19]	*	*	*	0	0	*	0	0	4
Clift 2018 [20]	*	*	*	0	0	*	0	0	4
Tsang 2018 [21]	*	*	*	0	0	*	0	0	4
Kowalsky 2021 [22]	*	*	*	0	*	*	0	0	5
Marks 2023 [23]	*	*	*	0	*	*	0	0	5

^ Adaquate follow up period was taken to be period of time necessary to calculate median survival. “*” Indicate quality present. “0” indicates quality not present.

## Data Availability

The original contributions presented in the study are included in the article/Supplementary Materials, further inquiries can be directed to the corresponding author.

## Supplementary Materials

The following supporting information can be downloaded at: https://www.mdpi.com/article/10.3390/diagnostics14161773/s1, Search strategy; Table S1. Tumour Characteristics.
