# Supplementary material for: The Optimal Approach to Surgical Management of Goblet Cell Carcinoid of the Appendix: A Systematic Review
Source: Diagnostics (Basel). 2024 Aug 14;14(16):1773. doi: 10.3390/diagnostics14161773 (PMC11353492; doi:10.3390/diagnostics14161773)
Supplement: Supplementary file 1 [file diagnostics-14-01773-s001.zip › diagnostics-3086872-supplementary.pdf]

Supplementary Material: The Optimal Approach to Surgical Management of Goblet Cell Carcinoid of the Appendix: A Systematic Review

Search strategy:
This included the following search terms and Boolean operators: ("goblet cell" OR "mucinous carcinoid" OR "adenocarcinoid") AND ("appendix" OR "appendiceal") AND ("surgical" OR "surgery" OR "operative" OR "operation" OR "intervention" OR "management" OR "treatment" OR "therapy" OR "therapeutic" OR "appendicectomy" OR "appendectomy" OR "colectomy" OR "hemicolectomy" OR "resection").

Results:
Table S1. Tumour Characteristics:

Tumour Location		Base [%]	Body [%]	Tip [%]
	Bucher 2005	2 [8]	1 [4]	4 [16]
	Clift 2018	10 [40]	5 [20]	3 [12]
	Total	12 [48]	6 [24]	7 [28]
Tumour size		Average (range)	Size Breakdown	
	Bucher 2005	Median 1.1 cm (0.7-3 cm)	-	
	Tsang 2018	-	<2cm (n=19), >/=2cm (n=2)	
Tumour Stage		Stage I (%)	Stage II (%)	Stage III (%)
	Clift 2018	1 (0.03)	10 (0.33)	5 (0.16)
	Kowalasky 2021	217 (7.07)	742 (24.19)	124 (4.04)
	Marks 2023	344 (11.21)	1237 (40.32)	191 (6.23)
	Total	562 (18.32)	1989 (64.83)	320 (10.43)
Tumour Grade		G1 [%]	G2 [%]	G3 [%]
	Clift 2018	3 [0.2]	6 [0.4]	9 [0.6]
	Kowalasky 2021	216 [14.5]	172 [11.5]	93 [6.2]
	Marks 2023	430 [28.9]	316 [21.2]	245 [16.4]
	Total	649 [43.6]	494 [33.1]	347 [23.3]
